# p.P1379S, a benign variant with reduced ATP7B protein level in Wilson Disease

**DOI:** 10.1002/jmd2.12127

**Published:** 2020-05-19

**Authors:** Fan Yi, Sheri A. Poskanzer, Candace T. Myers, Jenny Thies, Christopher J. Collins, Remwilyn Dayuha, Phi Duong, Roderick Houwen, Si Houn Hahn

**Affiliations:** ^1^ Seattle Children's Research Institute Seattle Washington USA; ^2^ Department of Pediatrics University of Washington, School of Medicine Seattle Washington USA; ^3^ Department of Laboratories Seattle Children's Hospital Seattle Washington USA; ^4^ Biochemical Genetics Seattle Children's Hospital Seattle Washington USA; ^5^ Wilhelmina Children's Hospital, Utrecht University Utrecht Netherlands

**Keywords:** ATP7B quantification, Benign variant, immuno‐SRM, newborn screening, p.P1379S, Wilson disease

## Abstract

**Background:**

Wilson disease (WD) is an autosomal recessive disorder of copper transport caused by inherited defects in the *ATP7B* gene and results in toxic accumulation of copper in various organs. We previously reported a family with three consecutive generations affected by WD that carries the variant, p.P1379S, which was classified at the time as likely pathogenic. However, recent investigations of the p.P1379S variant indicate a possible conflict of interpretations regarding its pathogenicity. This led us to explore the quantification of ATP7B in dried blood spots (DBS) using a surrogate peptide to study the effects of the p.P1379S variant on ATP7B concentrations in two unrelated families with the common p.P1379S variant.

**Methods and results:**

ATP7B was quantified using the peptide immunoaffinity enrichment coupled with selected reaction monitoring mass spectrometry (immuno‐SRM) method which utilizes antibody‐mediated peptide capture from DBS. Two patients affected with WD had undetectable ATP7B level while four compound heterozygous children with one known pathogenic variant and the p.P1379S had significantly reduced ATP7B levels. Of note, all four children remain asymptomatic without abnormal laboratory consequences despite being untreated for WD.

**Conclusion:**

These two families demonstrated that p.P1379S, when compounded with two known pathogenic variants, resulted in significantly reduced protein levels but retained enough function to maintain normal copper homeostasis. This implies that p.P1379S is benign in nature. A better understanding of the nature and consequences of variants in WD will help in informing patient care and avoiding unnecessary treatments.

**SYNOPSIS**

Utilization of immuno‐SRM analysis to determine the effect of the p.P1379S variant on ATP7B concentration in Wilson disease.

## 1 INTRODUCTION

Wilson disease (WD, OMIM #277900) is an autosomal recessive disorder resulting in copper accumulation due to biallelic pathogenic variants in the *ATP7B* gene (NM_000053.3) which encodes for ATP7B (EC # 7.2.2.8), a copper transporting P‐type ATPase. The estimated prevalence is 1 in 30 000 and a carrier frequency of 1 in 90 individuals with regional variations.^1^, ^2^ There are over 700 variants associated with *ATP7B* gene.^3^ The majority of the pathogenic variants that have been studied resulted in markedly decreased protein level of ATP7B due to the enhanced degradation, or absence or decay of mRNA.^3^, ^4^, ^5^, ^6^, ^7^, ^8^ Without functional ATP7B, copper accumulates in various organs resulting in a spectrum of liver disease, neurologic abnormalities, psychiatric manifestations, and the characteristic finding of Kayser‐Fleischer rings in the cornea.

Biochemical markers of WD include low serum ceruloplasmin, subnormal serum copper, high urinary copper excretion, and increased hepatic copper concentration in liver biopsy.^9^ Treatment is usually achieved by reducing the copper overload with chelation (penicillamine and trientine), pharmaceutical interference with gastrointestinal absorption (zinc), and restriction of dietary copper intake.^10^

We recently reported a method for using a signature peptide, ATP7B 1056, from ATP7B as a biomarker for WD.^11^ ATP7B quantification was achieved by peptide immunoaffinity enrichment coupled with selected reaction monitoring mass spectrometry (immuno‐SRM) which utilizes antibody‐mediated peptide capture from dried blood spots (DBS). The immuno‐SRM method can detect and quantify extremely low abundance proteins such as ATP7B from DBS samples at low pmol/L concentrations.^11^, ^12^

Our recent investigations of the c.4135C>T (p.P1379S) variant, previously reported as likely pathogenic, indicated a possible conflict of interpretation of pathogenicity.^13^ In our previously reported family case^14^ and an additional Dutch family reported here, all four compound heterozygous children carrying one known pathogenic variant and the p.P1379S variant remain asymptomatic even without treatment or dietary restrictions. Their biochemical and laboratory evaluations including 24‐hour urine copper studies all remained within the normal ranges. Here, we utilized Immuno‐SRM for ATP7B to investigate the effects of heterozygous p.P1379S variant on in vivo ATP7B concentrations in DBS from these families.

## 2 CASE REPORT

**FIGURE 1:**
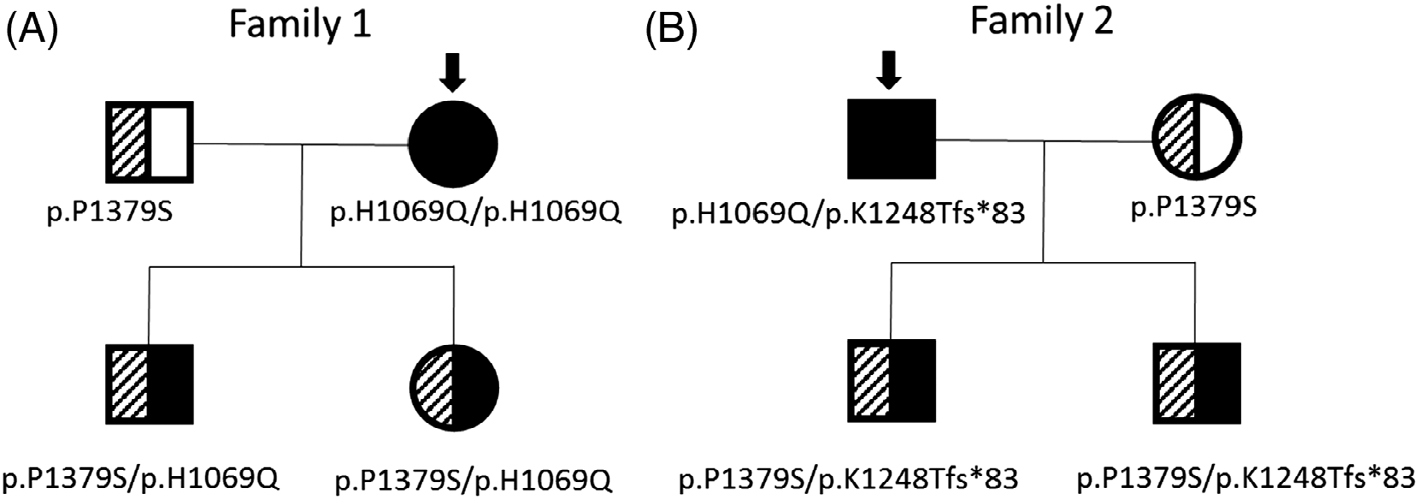
Pedigree of two unrelated families. (Arrow indicates parent with WD in the family)

The first family (family 1 in Figure 1A) has three consecutive generations affected by WD as previously reported.^14^ The proband was diagnosed in childhood and is homozygous for the most common pathogenic variant, c.3207C>A (p.H1069Q), in *ATP7B*. Her husband had carrier testing, which revealed heterozygosity for the variant, c.4135C>T (p.P1379S), classified at the time as likely pathogenic. Their two children were found to be compound heterozygous for both variants, p.P1379S and p.H1069Q, and were started on low dose zinc treatment in their early childhood.

Quantification of ATP7B from DBS revealed that the p.H1069Q homozygous mother had non‐detectable ATP7B level, while the carrier father showed an ATP7B concentration of 92.4 pmol/L. Their compound heterozygous son and daughter had concentrations of 26.0 and 24.7 pmol/L, respectively. These values are approximately 4 standard deviations (SD = 42.5 pmol/L) below the average of normal controls (192.5 pmol/L), while their heterozygous father is within 2.5 standard deviations (92.4 pmol/L). Both children have discontinued the zinc treatment for over 21 months without clinical or biochemical consequences: 24‐hour urine copper, liver transaminases, serum copper, and serum ceruloplasmin quantifications have remained within the normal range. Liver biopsy was not performed.

The second family (family 2 in Figure 1B) consists of a symptomatic father with WD, his partner, and their two children. The father is compound heterozygous for the variants, c.3741_3742dupCA (p.K1248Tfs*83), formerly known as c.3742_3743insCA (p.K1248Tfs*83),^15^ and c.3207C>A (p.H1069Q). The mother of the two children underwent *ATP7B* sequencing and was found to be heterozygous for the p.P1379S variant. Both of their sons, ages 14 and 5 years, are compound heterozygous for p.K1248Tfs*83 and p.P1379S.

The affected father had non‐detectable ATP7B, while the carrier mother had a concentration (118.9 pmol/L) within 2.5 SD of the average from normal control. The two compound heterozygous children had significantly reduced ATP7B concentrations of 24.2 and 14.5 pmol/L for the older brother and younger brother, respectively. Both children are asymptomatic with normal lab results and they have never received treatments for WD.

## 3 DISCUSSION

The most common pathogenic variant for *ATP7B*, p.H1069Q, has been known to cause reduced stability and rapid degradation of ATP7B,^16^, ^17^ which leads to a significant reduction of ATP7B level. The p.K1248Tfs*83 variant found in family 2 has only been observed twice in the heterozygous state with an allele frequency (AF) of 0.0007118% in gnomAD. This variant is most likely to be pathogenic due to the known consequences of frameshifting mutations.^18^ As expected, the ATP7B concentrations in the two patients homozygous or compound heterozygous for these mutations (Table 1) were not detectable in DBS.

**TABLE 1:** Genotypes and ATP7B peptide concentrations in two unrelated families

		Variant 1	Variant 2	ATP7B Peptide (pmol/L)
Family 1	Mother	p.H1069Q	p.H1069Q	ND
	Father	p.P1379S	Wt	92.4
	Child 1 (8 y)	p.H1069Q	p.P1379S	26.0
	Child 2 (6 y)	p.H1069Q	p.P1379S	24.7
Family 2	Father	p.K1248Tfs*83	p.H1069Q	ND
	Mother	p.P1379S	Wt	118.9
	Child 1 (14 y)	p.K1248Tfs*83	p.P1379S	24.2
	Child 2 (5 y)	p.K1248Tfs*83	p.P1379S	14.5

*Note*: ATP7B control, 192.5 ±42.5 (n = 100).

Abbreviations: ND, nondetectable; Wt, wild type.

When we initially published the case report for family 1,^14^ p.P1379S was considered as pathogenic based on previous report.^13^ However, this variant currently has been downgraded to a variant of uncertain significance (VUS) and has an AF in gnomAD (0.1684%, European 0.1877%, with one homozygote reported) that exceeds the most frequent pathogenic variants in *ATP7B*, p.H1069Q (AF 0.1111%). In addition, the p.P1379S variant demonstrated no deleterious effect on protein level in yeast, polarized hepatic cells and fibroblasts. In the yeast model, p.P1379S showed normal protein expression, copper transporting and oxidase activity.^19^ In polarized hepatic cells and fibroblasts, p.P1379S demonstrated normal tyrosinase activation, protein expression, and copper‐responsive trafficking.^4^ Altogether, this raised the possibility of conflicting interpretations regarding the pathogenicity of the p.P1379S variant. The rarity of compound heterozygosity of this variant in the patient cohort^20^ and the existence of one homozygous individual in the general population support that this is a likely benign variant. A pathogenic variant with this frequency would likely have been seen and reported with a much higher frequency than the p.H1069Q variant. However, in multiple studies of WD patient cohorts, p.H1069Q variant were observed in more than 20% of the patients while no p.P1379S variant was reported^21^, ^22^

These two families demonstrated that while p.P1379S results in reduced protein levels *in vivo*, the remaining ATP7B quantity and function is sufficient to maintain normal copper homeostasis in vivo, that is, normal serum copper and ceruloplasmin level, normal 24‐hour urine copper and liver enzymes without any treatments and dietary restrictions, up to 14 years of age. These studies support that the loss of function of ATP7B by this variant seems unlikely. As one of the key functional proteins for copper homeostasis, ATP7B accomplishes its role by performing several functions including copper‐induced translocation, copper exporting activity, and interaction with other proteins.^7^, ^23^ As discussed above, considering the in vitro performance of p.P1379S variant and the asymptomatic children with this variant, it is conceivable that the p.P1379S variant results in normal copper‐induced translocation, copper exporting activity, and copper incorporation into apoceruloplasmin. In contrast to the in vitro studies on p.P1379S, we do not know why the ATP7B protein is reduced in these cases. It is conceivable that misfolding of one variant may affect the other co‐translated product but will require further investigation to understand better about the protein interaction or assembly.

We previously proposed that the immuno‐SRM method for quantifying ATP7B concentration in DBS has the potential for newborn screening (NBS) application.^11^ Our study indicates that p.P1379S variant could potentially contribute to a false positive in compound hetertozygotes with severe pathogenic variants. Genetic analysis is hence necessary to confirm or exclude the diagnosis once the ATP7B peptide analysis showed the reduced protein level. Of note, the p.P1379S variant was recently reported as one of the five most frequent known WD variants by meta‐analysis.^24^ Incorrect information or lack of understanding about certain variants can mislead the interpretation of genetic analyses. Our observation therefore is important to consider to avoid any unnecessary treatment in suspected or presymptomatic WD patients.

## CONFLICT OF INTEREST

Dr. Si Houn Haun has received compensation and sponsored travel from Alexion Pharmaceutical Company. All other authors have no conflict of interest.

## AUTHOR CONTRIBUTIONS

Fan Yi was involved in the study design, peptide analysis performance, preparing, drafting, and revising the manuscript for publication. Sheri A. Poskanzer and Jenny Thies were involved in the acquisition of clinical patient data, drafting and revising the manuscript, creating and editing the table and figure. Christopher J. Collins was involved in study design, peptide analysis performance, and revising the manuscript. Candace T. Myers was involved in performing molecular testing and interpretation. Remwilyn Dayuha was involved in sample preparation and revising the manuscript. Phi Duong was involved in IRB preparation, consent, and revising the manuscript. Si Houn Hahn conceptualized and designed the study and was involved in data analysis, interpretation, drafting, and revising the manuscript. All authors revised the manuscript critically for important intellectual content and gave final approval of the version to be published. All authors agreed to be accountable for all aspects of the work in ensuring that questions related to the accuracy or integrity of any part of the work are appropriately investigated and resolved.

## ETHICS APPROVAL

The study has been approved by the Institutional Review Board (IRB) for human subject research (IRB# 15194). Informed consent was obtained from all patients for being included in the study.
